# The Inflammatory Response to Alcohol Consumption and Its Role in the Pathology of Alcohol Hangover

**DOI:** 10.3390/jcm9072081

**Published:** 2020-07-02

**Authors:** Aurora J.A.E. van de Loo, Marlou Mackus, Oran Kwon, Illathu Madhavamenon Krishnakumar, Johan Garssen, Aletta D. Kraneveld, Andrew Scholey, Joris C. Verster

**Affiliations:** 1 Division of Pharmacology, Utrecht Institute for Pharmaceutical Sciences (UIPS), Utrecht University, 3584CG Utrecht, The Netherlands; a.j.a.e.vandeloo@uu.nl (A.J.A.E.v.d.L.); marloumackus@gmail.com (M.M.); j.garssen@uu.nl (J.G.); a.d.kraneveld@uu.nl (A.D.K.); 2 Institute for Risk Assessment Sciences (IRAS), Utrecht University, 3584CM Utrecht, The Netherlands; 3 BioFood Laboratory/BioFood Network, Department of Nutritional Science and Food Management, Ewha Womans University, Seoul 120-750, Korea; orank@ewha.ac.kr; 4 Akay Natural Ingredients Private Limited, Ambunad Malaidumthuruthu, Cochin, Aluva, Kerala 683561, India; krishnakumar.im@Akay-group.com; 5 Global Centre of Excellence Immunology, Nutricia Danone Research, 3584CT Utrecht, The Netherlands; 6 Centre for Human Psychopharmacology, Swinburne University, VIC 3122 Melbourne, Australia; andrew@scholeylab.com

**Keywords:** alcohol, hangover, ethanol, acetaldehyde, acetate, oxidative stress, malondialdehyde, 8-isoprostane, cytokines, C-reactive protein

## Abstract

An increasing number of studies are focusing on the inflammatory response to alcohol as a potentially important determinant of hangover severity. In this article, data from two studies were re-evaluated to investigate the relationship between hangover severity and relevant biomarkers of alcohol metabolism, oxidative stress and the inflammatory response to alcohol. Hangover severity was significantly and positively correlated with blood concentrations of biomarkers of the inflammatory response to alcohol, in particular, Interleukin-6 (IL-6), tumor necrosis factor-alpha (TNF-α) and C-reactive protein (CRP). At 4 h after alcohol consumption, blood ethanol concentration (but not acetaldehyde) was significantly and positively associated with elevated levels of IL-6, suggesting a direct inflammatory effect of ethanol. In addition, biomarkers of oxidative stress, i.e., malondialdehyde and 8-isoprostrane, were significantly correlated with hangover severity, suggesting that oxidative stress also contributes to the inflammatory response. The timing of the assessments suggests initial slow elimination of ethanol in the first hours after alcohol consumption. As a consequence, more ethanol is present in the second half of the night and the next morning, which will elicit more oxidative stress and a more profound inflammatory response. Together, these processes result in more severe hangovers.

## 1. Introduction

The alcohol hangover refers to the combination of negative mental and physical symptoms, which can be experienced after a single episode of alcohol consumption, starting when blood alcohol concentration (BAC) approaches zero [1,2]. Reviews on the pathology of alcohol hangover [3,4,5] suggest that, although research is limited, both alcohol metabolism and the immune system play an important role in the development of alcohol hangover. The role of alcohol metabolism and oxidative stress are discussed elsewhere in detail [6]. These analyses revealed that higher blood concentrations of ethanol (but not acetaldehyde) and oxidative stress (a combination between depletion of antioxidants and an increase in free radicals, originating in the liver) were associated with having more severe hangovers. These processes are determined by the ethanol elimination rate. In other words, fast elimination of ethanol was associated with experiencing less severe hangovers [7]. The current analysis aimed to further elaborate the role of immune responses elicited by alcohol consumption in the development of the alcohol hangover.

A possible role of the immune system in the pathology of alcohol hangover was first hypothesized in the 1980s [8,9]. Kaivola et al. [8] examined the effects of tolfenamic acid on alcohol hangover in *n* = 30 social drinkers. Tolfenamic acid is a non-steroidal anti-inflammatory drug (NSAID), and prostaglandin (PG) is an inhibitor used for the treatment of pain, and as such, it could be hypothesized that the drug would be helpful in mitigating related hangover symptoms. From *n* = 10 subjects, blood samples were also taken, and prostaglandin PGE_2_, PGF_2α_, thromboxane B_2_ (TXB_2_) and 6-keto PGF_1α_ were determined at 0, 5, 10 and 20 h after drinking. Subjects rated the efficacy of the drug on an 11-point scale ranging from “very ineffective” to “very effective”, with subjects rating the treatment as significantly more effective than placebo. The same study further showed a significant reduction in the severity of several hangover symptoms after drug administration, with headache, dry mouth and thirst being the most drug-sensitive items. Alcohol consumption significantly increased PGE_2_ and TXB_2_ concentrations (the placebo condition). Administering tolfenamic acid partly counteracted the observed increments. No significant changes in PGF_2α_ and 6-keto PGF_1α_ were observed over time, and tolfenamic acid had no significant effect on these assessments.

Paratainen [9] reviewed the evidence for the role of prostaglandins in hangover, which at that time was limited to the study by Kaiviola et al. [8]. The review concluded that alcohol has an effect on prostaglandin synthesis, which can be counteracted by a prostaglandin inhibitor such as tolfenamic acid. It took 20 years for other studies to confirm that alcohol consumption is accompanied by an immune response, and that elevated levels of cytokines in blood and saliva may be related to the presence and severity of the alcohol hangover.

Kim et al. [10] demonstrated an inflammatory response accompanying alcohol consumption that was significantly associated with next-day hangover severity. In their study, twenty male Asian subjects had an evening of alcohol consumption (soju, a distilled beverage of Korean origin), which was compared to an alcohol-free control day. The following morning, blood samples were collected for determination of cytokine concentrations. The hangover scale consisted of two subscales, computing sum scores of ratings of subjective hangover symptoms (i.e., thirst, tension, depression and general discomfort, rated by the participant) or somatic hangover symptoms (i.e., paleness, tremor, perspiration, nystagmus, vomiting and general appearance, rated by two psychiatrists). On the hangover day, compared to the alcohol-free control day, significantly increased blood concentrations were found for interleukin (IL)-10, IL-12 and interferon-gamma (IFN-γ), whereas no significant changes were observed for IL-1β, IL-4, IL-6 or tumor necrosis factor-alpha (TNF-α). Furthermore, the increase in IL-12 and IFN-γ correlated significantly with overall hangover severity. Significant correlations with subjective hangover scale scores were found for difference scores (Δ, alcohol-control day) Δ IFN-γ, and significant correlations with somatic hangover scale scores were found for Δ TNF-α and Δ IL-12.

Wiese et al. [11] examined the effects of *Opuntia ficus indica* (OFI) (prickly pear) on alcohol hangover, administered 5 h before alcohol consumption. OFI is used as a dietary supplement for its antioxidant and anti-inflammatory properties [12]. The authors concluded that the alcohol hangover is mediated by an inflammatory response, as they observed significant correlations between blood C-reactive protein (CRP) concentration and hangover severity. After placebo treatment, CRP levels rose by 50%, accompanied by experiencing an alcohol hangover. After administering OFI, no inflammatory response was seen (CRP levels did not differ from placebo) and several hangover symptoms were alleviated.

George et al. [13] examined the effects of a new hangover treatment (*Phyllanthus amarus*, PHYLLPROTM, 750 mg/day, administered for 10 days) on hangover severity. *Phyllanthus amarus* is traditionally used in India for the treatment of hepatitis and jaundice, and for general liver health, and its reported effectiveness is most likely due to its antioxidant properties [14]. George et al. assessed a variety of blood cytokines the day following alcohol consumption, and hangover severity reported 10 h after drinking. In this study, IL-8, IL-10 and IL-12p70 levels were increased significantly in the active group, denoting an anti-inflammatory effect of the treatment compared to the placebo treatment alcohol challenge. Unfortunately, the authors did not report correlations between inflammatory markers and the presence or severity of alcohol hangover.

Van de Loo et al. [15] examined cytokine concentration in saliva. *n* = 36 healthy social drinkers (18 to 30 years old) participated in a naturalistic study. Based on lifetime self-report, at screening, subjects were allocated to a hangover-sensitive group (those who reported having hangovers), or the hangover-resistant group (those reporting never having hangovers despite consuming large quantities of alcohol). The morning following an evening of alcohol consumption and an alcohol-free control day, saliva samples were collected, and cytokine concentrations were assessed. Significant increases in IL-6 and IL-10 concentrations were observed the morning after heavy drinking. No significant changes were found for IL-1β, IL-8 and TNF-α. Interestingly, no significant differences were observed between the hangover-sensitive group and the drinkers that claimed to be hangover-resistant. The elevation in saliva IL-6 and IL-10 concentration did not significantly correlate with overall hangover severity. However, the correlations between IL-6 and headache (r = 0.572, *p* = 0.017) and between IL-6 and concentration problems (r = 0.536, *p* = 0.027) showed a trend towards significance (taking into account multiple comparisons, a conservative *p*-value cut-off was applied for statistical significance, which was set at *p* < 0.002).

Ethanol may elicit an inflammatory response directly, or indirectly via its breakdown products and oxidative stress [6]. The conversion of ethanol into acetaldehyde involves the production of reactive oxygen species (ROS) and reactive nitrogen species (RNS), which are both harmful for the body and thus elicit an immune response [16]. The free radicals are usually neutralized by antioxidants such as superoxide dismutase or glutathione. Oxidative stress refers to the situation when the amount of ROS and RNS is much larger than the present antioxidants. The free radicals are highly reactive. For example, malondialdehyde–acetaldehyde adducts (MAA adducts) are formed, which have proinflammatory properties [17]. The MAA adducts are recognized by the body as foreign substances, and as a result, an immune response is elicited [18,19,20], including increased secretion of cytokines and chemokines.

Other important biomarkers of oxidative stress are isoprostanes, i.e., prostaglandin-like compounds formed via non-enzymatic free radical-initiated lipid peroxidation of arachidonic acid, and other polyunsaturated fatty acids (PUFAs), including α-linolenic acid, eicosapentaenoic acid (EPA), adrenic acid and docosahexaenoic acid (DHA) [21]. However, studies that have examined oxidative stress in human subjects in the context of alcohol hangover are scarce [6].

Together, previous studies demonstrated that alcohol consumption is followed by an inflammatory response. However, the precise mechanism of how the inflammatory response is elicited is unclear, and there are mixed results on which specific cytokines play a key role in this process. Further, it is uncertain to what extent changes in cytokine concentrations correlate significantly to self-reported hangover severity. Finally, the previous studies also do not provide insight into how cytokine changes are related to biomarkers of alcohol metabolism and oxidative stress. This knowledge is, however, vital to better understand the pathology of the alcohol hangover. Therefore, the aim of the current article was to further investigate and integrate data on alcohol metabolism, oxidative stress and the inflammatory response to alcohol consumption in relation to alcohol hangover. To this extent, we re-evaluated data from the two available studies that collected this combined data in the same subjects, enabling a direct investigation into their interrelationships [22,23].

## 2. Methods

Data from two studies were re-evaluated (see original articles for details on methodology) [22,23].

In the first double-blind crossover study, Kim et al. [22] examined the effects of the fruit of *Hovenia dulcis* versus placebo on hangover severity in *n* = 26 healthy men (mean ± standard deviation (SD) age of 23.7 ± 0.3 years old), with heterozygous ALDH2. Subjects consumed 360 mL of Korean Soju (50 g alcohol) together with Hovenia dulcis extract (2460 mg) or matched placebo. Blood samples were taken 1, 4 and 12 h after alcohol consumption and ethanol and acetaldehyde concentrations were determined. Blood cytokine concentrations of IL-6, IL-10, IL-12 and TNF-α were assessed at 4 and 12 h after drinking alcohol. Hangover severity was assessed the next morning, 12 h after alcohol consumption, using a 14-item scale on which symptoms could be scored on a 5-point likers scale, which was developed for this study. The sum score of the 14 items was used as the overall hangover severity score. The study was conducted in accordance with the Declaration of Helsinki and was approved by the Institutional Review Board of Chonbuk National University Hospital (World Health Organization International Clinical Trials Registry Platform identification number: KCT0001626).

In a second double-blind crossover study, Mammen et al. [23] examined the effect of a polyphenolic extract of clove buds (Clovinol) versus placebo on hangover severity in *n* = 16 healthy men (mean ± SD age of 29.5 ± 6.2 years old). The study was conducted in India, and genetic polymorphisms in alcohol metabolic enzymes were not considered. Blood ethanol and acetaldehyde concentration were determined before and at 0.5, 2, 4 and 12 h after alcohol consumption (240 mL of 42.8% McDowell’s Very Superior Old Pale (V.S.O.P.) Brandy; United Spirits Limited, Bangalore, India). To investigate the inflammatory response, IL-6 and CRP were also assessed at the same time points. Finally, two antioxidants (glutathione and superoxide dismutase (SOD)), and two biomarkers of oxidative stress (8-isoprostane and malondialdehyde) were measured. A hangover scale was completed 14 h after drinking. Hangover severity was measured using a 15-item scale on which symptoms could be scored on scales ranging from 1 (absent) to 10 (extreme). The scale was composed by the authors. The average score of the 15 items was computed and used as overall hangover severity score.

The study was carried out in accordance with the clinical research guidelines established by the Government of India, and protocol was approved by an independent ethical committee (Clinical trial Reg. No. ECR/64/Indt/KA/2013).

### Statistical Analysis

Statistical analyses were conducted with SPSS, version 25 (Armonk, IBM Corp, New York, NY, USA). Subjects were only included in the current analysis if they had hangover severity scores greater than zero. Regarding individual data, outliers (mean ± 3 interquartile ranges) were omitted from the analysis. Only data from the placebo condition (i.e., alcohol-only, without treatment) were used. Means and standard deviation (SD) were computed for each variable. Non-parametric Spearman’s correlations were used to account for a non-normal distribution.

Due to the relatively small sample size, a bootstrapping technique was applied [24,25] to simulate the population distributions of the partial correlations (r_P_). To obtain an adequate resampling size [26], B = 10,000 bootstrapped samples (of *n* = 8 subjects each) were created by randomly drawing cases (resampling), with replacement, from the original sample. For each of the bootstrap samples, the bootstrapped partial correlation, denoted as r_PB_, was calculated. The standard error (SE) represents how much the r_PB_s vary across the bootstrap samples, and the reported ‘bias’ measure represents the deviation of the overall r_PB_ from the r_P_ that was obtained from the original sample [27]. The bias-corrected and accelerated bootstrapped 95% confidence interval (BCa 95% CI_B_) was computed for each correlation [28]. The lower and upper limit of the BCa 95% CI_B_ can range from −1 to +1, with narrower BCa 95% CI_B_’s implying greater precision. Bootstrapping does not provide *p*-values, but significance is determined by the BCa 95% CI_B_. If the BCa 95% CI_B_ does not include zero, the r_PB_ is considered statistically significant (corresponding to a significance level of α = 5%).

## 3. Results

### 3.1. Study 1

**Figure 1:**
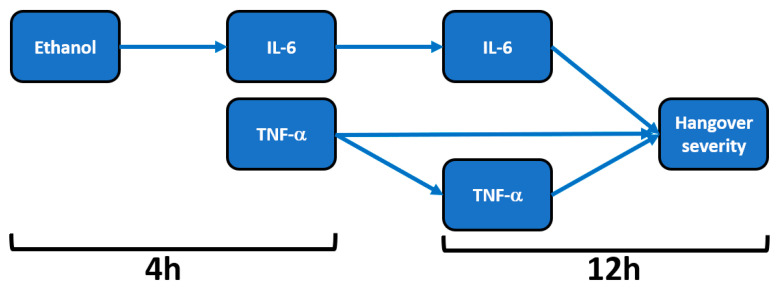
Relationship of hangover severity with blood ethanol concentration and the inflammatory response to alcohol. Note: Arrows represent significant correlations after bootstrapping. Abbreviations: Il = interleukin, TNF = tumor necrosis factor, h = hours after alcohol consumption. Data from Kim et al. [22].

Only data from the placebo condition (i.e., alcohol-only, without treatment) were considered. Analysis of the raw data revealed that *n* = 10 subjects did not report any hangover symptoms. Data from these *n* = 10 subjects were omitted. The results from the remaining *n* = 16 subjects are summarized in Table 1 and Figure 1.

Mean (SD) hangover severity, assessed 12 h after alcohol consumption, was 16.2 (1.8). The analysis revealed no significant correlations between blood ethanol and overall hangover severity at any timepoint. Also, no significant correlations were found between blood acetaldehyde and overall hangover severity at any timepoint. Significant correlations were found between hangover severity and TNF-α at 4 h (r_B_ = 0.679, BCa 95%CI_B_ = 0.440, 0.835) and TNF-α at 12 h (r_B_ = 0.625, BCa 95%CI_B_ = 0.252, 0.889). A significant correlation was also found between IL-6 at 12 h and hangover severity (r_B_ = 0.459, BCa 95%CI = 0.036, 0.796).

On the contrary, the inflammatory response seems to be directly related to blood ethanol concentrations. That is, significant positive correlations were found between blood ethanol at 4 h and IL-6 at 4 h (r_B_ = 0.482, BCa 95%CI_B_ = 0.038, 0.803) and between blood ethanol at 4 h and IL-6 at 12 h (r_B_ = 0.573, BCa 95%CI_B_ = 0.216, 0.835). Correlations between IL-6 and TNF-α did not reach statistical significance for any timepoint combination. In contrast to ethanol, the inflammatory response was not significantly associated with acetaldehyde concentrations.

### 3.2. Study 2

Data on hangover severity of three subjects were not collected. Raw data from the remaining *n* = 13 subjects were used for the statistical analysis. Their mean (SD) hangover severity, assessed 14 h after alcohol consumption, was 4.4 (0.5). The results of the assessments in the alcohol-only condition are summarized in Table 2.

Acetaldehyde levels significantly increased directly after alcohol consumption (*p* < 0.0001), but declined steadily thereafter, and at 12 h after drinking, acetaldehyde could no longer be detected, whereas ethanol concentrations were still detectible after this time period. No significant correlations were found between hangover severity and blood ethanol or acetaldehyde concentrations at any time point after alcohol consumption.

CRP concentration at 4 h was significantly associated with hangover severity at 14 h (r_B_ = 0.534, BCa 95%CI_B_ = 0.120, 0.825). No significant correlations between IL-6 and hangover severity were found at any time point, and the associations between blood ethanol and acetaldehyde concentrations and IL-6 were also not significant at any timepoint. A significant correlation was found between IL-6 at 2 h and CRP at 4 h (r_B_ = 0.587, BCa 95%CI_B_ = −0.874, −0.130). CRP concentrations did not correlate significantly with blood ethanol or acetaldehyde concentrations at any timepoint.

With regard to antioxidants, the assessments revealed that both glutathione and SOD concentrations showed a decrease after alcohol consumption, but at no timepoint did their concentrations correlate significantly with hangover severity. The concentration of 8-isoprostane increased over time and reached its highest point 12 h after alcohol consumption (*p* < 0.0001).

**Figure 2:**
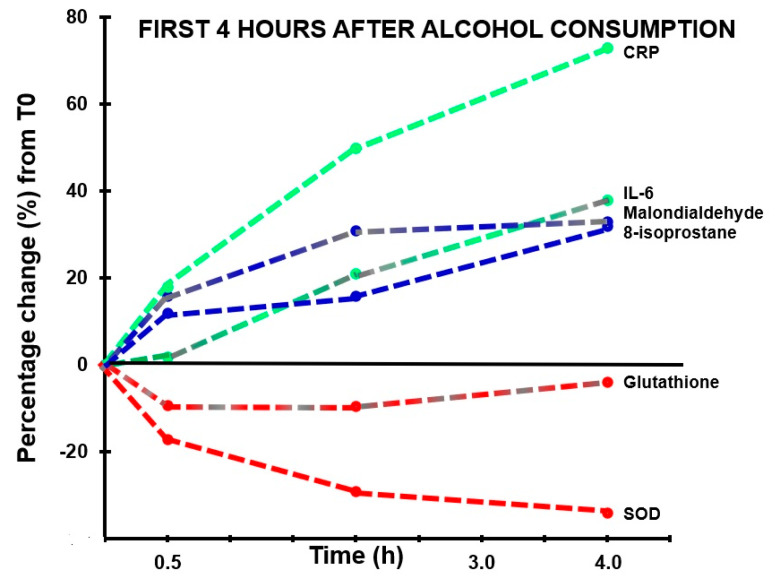
Markers of oxidative stress and inflammatory responses in the first four hours after alcohol consumption. Percentage changes between assessments made in the first 4 h after alcohol consumption relative to T0 (the assessment made before alcohol consumption) are shown. Abbreviations: CRP = C-reactive protein, IL-6 = interleukin-6, SOD = superoxide dismutase.

**Figure 3:**
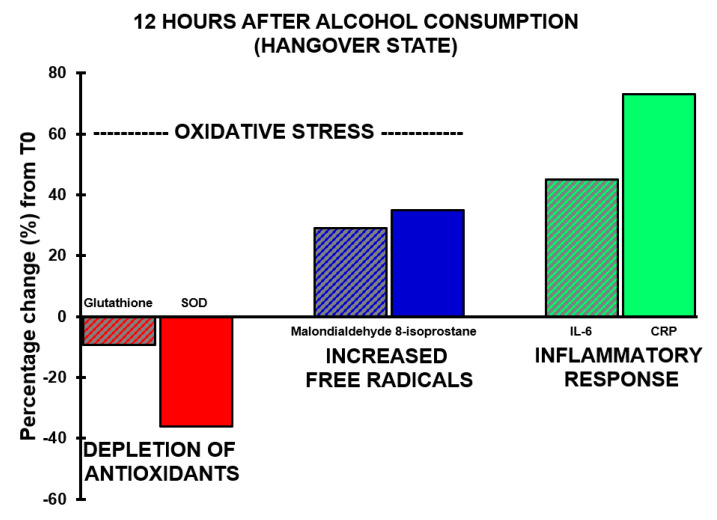
Oxidative stress and the inflammatory response during alcohol hangover. Percentage changes between assessments made 12 h after alcohol consumption relative to T0 (the assessment made before alcohol consumption) are shown. Abbreviations: CRP = C-reactive protein, IL-6 = interleukin-6, SOD = superoxide dismutase.

A significant correlation was found between hangover severity and 8-isoprostane at 12 h (r_B_ = 0.475, BCa 95%CI_B_ = 0.06, 0.78). Also, the blood malondialdehyde concentration at 0.5 h correlated significantly with hangover severity at 14 h (r_B_ = −0.707, BCa 95%CI_B_ = −0.96, −0.16). The negative correlation suggests that higher malondialdehyde concentrations directly after drinking are associated with experiencing less severe hangovers. 8-isoprostane at 2 h after alcohol consumption correlated significantly, and negatively, with acetaldehyde assessed 0.5 h (r_B_ = −0.612, BCa 95%CI_B_ = −0.884, −0.192) and 2 h (r_B_ = −0.613, BCa 95%CI_B_ = −0.881, −0.191) after alcohol consumption, while ethanol concentrations revealed non-significant positive associations at these timepoints. Assessments at 2 h after alcohol consumption revealed a significant negative association (r_B_ = −0.541, BCa 95%CI_B_ = 0.113, 0.833) between oxidative stress (8-isoprostane) and antioxidant depletion (glutathione). The results are summarized in Figure 2 and Figure 3.

## 4. Discussion

Multiple lines of evidence, as summarized elsewhere [6], suggest that the amount of ethanol present in the blood is an important determinant of hangover severity. Specifically, faster conversion of ethanol into acetaldehyde and other aldehydes is associated with having less severe hangovers [7]. Data from the two studies evaluated here confirm and extend previous findings. For example, the data support previously reported relationships between oxidative stress and inflammatory responses to alcohol consumption (i.e., increases in IL-6 and CRP). Blood malondialdehyde concentration at 0.5 h correlated negatively with hangover severity at 14 h. Thus, higher oxidative stress in the first hours after drinking was associated with less severe next-day hangovers. The significant positive correlation between hangover severity and 8-isoprostane at 12 h after alcohol consumption suggests that more oxidative stress, experienced at a later stage after alcohol consumption, is associated with having more severe hangovers. In other words, a quick ethanol metabolism results in more oxidative stress in the first hours of drinking. This is associated with having less severe next-day hangovers. On the other hand, if ethanol metabolism is relatively slow, there will be more oxidative stress the following morning, which is associated with having more severe hangovers. Increased ethanol concentration, but not acetaldehyde, was associated with elevated IL-6 levels. The magnitude of the inflammatory response correlated significantly with hangover severity.

Low-grade inflammation, i.e., relative minor elevations in proinflammatory cytokine concentrations, is a common underlying condition in many diseases [29]. Alcohol consumption is one of the many factors that can provoke such an elevation of proinflammatory cytokines. The studies reviewed in the introduction, and the data from the two studies presented here, demonstrate significant and robust elevations in cytokine concentrations, including IL-6, IL-10, IL-12, IFN-γ and TNF-α, after alcohol consumption and during the hangover state. The relationship between CRP and cytokine production is complex and bi-directional, and CRP production is usually followed in time by cytokine production, particularly IL-6 and TNF-α are induced by the presence of CRP [30].

Results of the two studies were in agreement that an inflammatory response follows alcohol consumption. However, there were also differences. For example, whereas Kim et al. [22] found a significant correlation between Il-6 and hangover severity, this correlation did not reach significance in the study by Mammen et al. [23]. Other studies also reported no significant correlation between IL-6 concentration and hangover severity [10,14]. The finding by Mammen et al. [23] that CRP concentration did correlate significantly with hangover severity is in line with previous findings [11]. In contrast to other studies [10,15], we did not find significant correlations between hangover severity and blood ethanol concentration. The observed differences between the outcomes of the two studies and available literature can be explained by differences in the study designs. For example, with regards to subject recruitment, one study took genetic polymorphism into account [22] whereas the other study did not [23]. The administered type and amount of alcohol differed between the two studies, which resulted in different blood alcohol concentrations. At 4h after alcohol consumption, the difference in blood alcohol levels was roughly 32.1 mg/dL (~0.03%) between the two studies, which might explain why some associations with overall hangover severity were only significant in one study. Further, different hangover assessment scales were used, and the assessments of hangover severity were made at different points in time after drinking (12 versus 14 h after drinking). Similarly, the biomarkers of alcohol metabolism and oxidative stress were assessed at various points in time after alcohol intake, and the timing of the assessments differed between the two studies. Of note, the expression of various cytokines is interrelated and time-dependent. Therefore, different timing of assessments may explain why in some study designs, a relationship of a biomarker of alcohol metabolism or specific cytokines significantly correlated with hangover severity, while in another study, the same correlation does not reach statistical significance. Notwithstanding this, it is clear from all studies that alcohol consumption is followed by oxidative stress and an inflammatory response.

There are several issues that deserve attention when interpreting the results of the two studies we re-analyzed here. First, participants of the studies were young males only. Therefore, it is unclear to what extent the results can be generalized to females and other age groups. Research has shown that age and sex differentially impact both alcohol elimination rate [31] and inflammatory responsiveness [32]. Other studies that did include both male and female subjects (discussed in the introduction section of this article) did not report any sex or age differences in aspects of the pathology of the alcohol hangover. However, these aspects may not have been reported as the investigated samples were not powered to conduct such comparisons. Future research should therefore be conducted in samples comprising both men and women, and also investigate other age groups. These studies should have sufficient sample size to allow comparisons among groups.

Second, the subjects in the re-evaluated studies were of Asian descent. There are differences reported in ethanol metabolism between different ethnic groups. For example, it has been found that in populations of Asian descent, subjects with ALDH2*2 alleles, i.e., those who breakdown acetaldehyde more slowly, typically report significantly worse hangovers, and are more likely to experience hangovers at relatively lower alcohol consumption levels. [33,34]. Therefore, future studies should confirm the current observations in subjects of non-Asian descent.

Third, the administered amount of alcohol consumed was relatively low in the re-evaluated studies. It would be interesting to further investigate the observed effects when higher amounts of alcohol are consumed that may better reflect real-life alcohol consumption. On the other hand, also with the currently applied consumption levels, inflammatory effects were evident. Moreover, recent studies have demonstrated that consuming large amounts of alcohol is not a necessity for having hangovers [35]. In fact, a recent large sample study revealed an average estimated BAC of 0.03% of subjects that reported a hangover [36], which is lower that the achieved BACs in the re-evaluated studies here.

Fourth, both studies assessed hangover severity using a composite scale of hangover symptoms. Recent examinations revealed that the magnitude of hangover severity of composite scales ultimately depends on which symptoms are included in such scales [37]. This may have had an impact on the correlational analyses. Future research should also use single-item hangover severity assessments as these better reflect overall hangover severity, and its impact. It is also important to thoroughly investigate whether potential participants of hangover studies will likely have a hangover when a certain amount of alcohol is consumed. In the study by Kim et al. [22], *n* = 10 subjects were excluded after participation because they reported no hangover during the study. Instead of excluding them after participation, it is more ideal to identify them during screening, and screening tools for this purpose are currently under development.

To conclude, it should be noted that the current body of data examining the pathology of the alcohol hangover is limited, and much more research is needed to further elucidate the exact nature of how ethanol metabolism, oxidative stress and the immune response are interrelated and may impact hangover severity. In this context, it is important to note that the two studies in this article comprised only male participants. As it is known that ethanol elimination rate is influenced by sex, future studies should be conducted to further investigate the role of the immune system on hangover in samples comprising both men and women, of different ethnicity and age.

**Figure 4:**
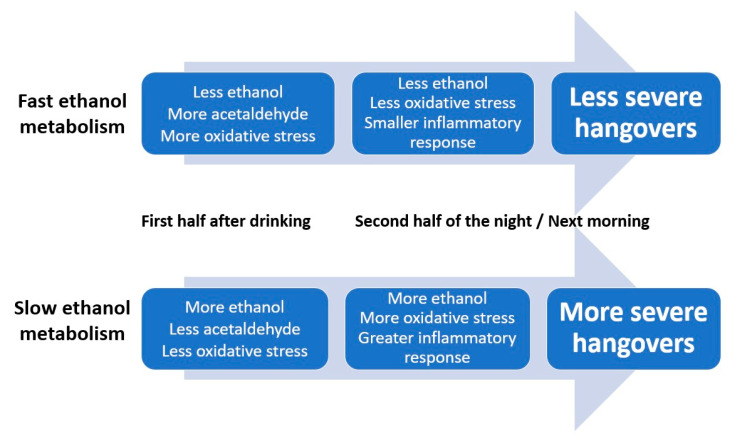
The relationship of alcohol metabolism, oxidative stress and the inflammatory response with hangover severity. Note: The rate of alcohol metabolism, i.e., the conversion of ethanol into acetaldehyde and other aldehydes, seems to be the primary determinant of hangover severity.

Taken together, the timing of the observed assessments (see Figure 2 and Figure 3) suggests that initial slow elimination of ethanol in the first hours after drinking results in more ethanol present in the second half of the night and the next morning, which will elicit more oxidative stress and a stronger inflammatory response. Together, these processes result in having more severe next-morning hangovers (See Figure 4).

## Figures and Tables

**Table 1:** Biomarkers of alcohol metabolism, the inflammatory response to alcohol consumption and their relation to hangover severity.

Biomarker	Time After Alcohol Consumption			
	0 h	1 h	4 h	12 h
Ethanol (mg/dL)	0.0 (0.0)	56.8 (10.0)	38.9 (7.2)	1.1 (0.4)
Acetaldehyde (mg/dL)	0.0 (0.0)	0.13 (0.07)	0.04 (0.02)	0.01 (0.01)
Interleukin (IL)-6 (pg/mL)	0.50 (1.2)	–	0.97 (1.7)	1.03 (1.7) *
Interleukin (IL)-10 (pg/mL)	0.35 (0.5)	–	0.29 (0.4)	0.28 (0.4)
Interleukin (IL)-12 (pg/mL)	0.04 (0.07)	–	0.01 (0.006)	0.01 (0.006)
Interferon-gamma (IFN-γ) (pg/mL)	0.09 (0.2)	–	0.02 (0.01)	0.04 (0.07)
Tumor necrosis factor-alpha (TNF-α) (pg/mL)	3.56 (1.9)	–	3.31 (1.4) *	4.00 (2.0) *

Biomarker assessments after consumption of alcohol to reach a desired breath alcohol concentration (BrAC) of 0.06%. Mean and standard deviation (SD) (between brackets) are shown. Significant correlations with hangover severity (*p* < 0.05), after bootstrapping (B = 10,000 bootstrapped samples), are indicated by *. – = not assessed. Data from Kim et al. [22].

**Table 2:** Biomarkers of alcohol metabolism, oxidative stress and the inflammatory response to alcohol consumption and their relation to hangover severity.

Biomarker	Time After Alcohol Consumption				
	0 h	0.5 h	2 h	4 h	12 h
Ethanol (mg/dL)	–	26.2 (4.5)	14.8 (3.5)	6.8 (1.0)	2.7 (0.4)
Acetaldehyde (mg/dL)	–	0.42 (0.03)	0.28 (0.05)	0.17 (0.02)	–
Interleukin (IL)-6 (pg/mL)	4.2 (0.0)	4.3 (0.8)	5.1 (1.0)	5.8 (1.0)	6.1 (1.0)
C-reactive protein (mg/L)	2.2 (0.6)	2.6 (0.7)	3.3 (0.8)	3.8 (0.7) *	3.8 (1.2)
8-isoprostane (pg/mL)	22.6 (4.5)	25.3 (3.5)	26.2 (4.8)	29.8 (7.1)	30.6 (6.5) *
Malondialdehyde (nmol/dL)	72.0 (7.3)	83.4 (7.2) *	94.4 (7.0)	95.5 (7.0)	92.6 (13.3)
Glutathione (nmol/mg Hb)	23.9 (3.4)	21.7 (3.1)	21.6 (3.9)	23.0 (2.4)	21.7 (3.5)
Superoxide dismutase (U/mL)	0.42 (0.1)	0.35 (0.1)	0.30 (0.1)	0.28 (0.1)	0.27 (0.1)

Biomarker assessments after consumption of alcohol to reach a desired BrAC of 0.03%. Mean and standard deviation (between brackets) are shown. Significant correlations with hangover severity (*p* < 0.05), assessed 14 h after alcohol consumption, are indicated by *. – = ethanol and acetaldehyde were not assessed at 0 h, and acetaldehyde could not be detected 12 h after alcohol consumption. Data from Mammen et al. [23].
